# Ovine recombinant PrP as an inhibitor of ruminant prion propagation *in vitro*

**DOI:** 10.1080/19336896.2017.1342919

**Published:** 2017-06-30

**Authors:** Rob G. Workman, Ben C. Maddison, Kevin C. Gough

**Affiliations:** a School of Veterinary Medicine and Science, The University of Nottingham, Sutton Bonington, Leicestershire, UK; b ADAS, The University of Nottingham, Sutton Bonington, Leicestershire, UK

**Keywords:** BSE, PMCA, prion diseases, protein inhibitors, recombinant PrP, Scrapie, therapeutics

## Abstract

Prion diseases are fatal and incurable neurodegenerative diseases of humans and animals. Despite years of research, no therapeutic agents have been developed that can effectively manage or reverse disease progression. Recently it has been identified that recombinant prion proteins (rPrP) expressed in bacteria can act as inhibitors of prion replication within the *in vitro* prion replication system protein misfolding cyclic amplification (PMCA). Here, within PMCA reactions amplifying a range of ruminant prions including distinct *Prnp* genotypes/host species and distinct prion strains, recombinant ovine VRQ PrP displayed consistent inhibition of prion replication and produced IC50 values of 122 and 171 nM for ovine scrapie and bovine BSE replication, respectively. These findings illustrate the therapeutic potential of rPrPs with distinct TSE diseases.

## INTRODUCTION

Prion diseases, also known as transmissible spongiform encephalopathies (TSEs), are fatal neurodegenerative diseases that affect humans and animals. Examples include scrapie in sheep, bovine spongiform encephalopathy (BSE) in cattle and Creutzfelt Jakob disease (CJD) in humans. The central event in these diseases is the conversion of cellular prion protein (PrP^C^) into the pathogenic isoform PrP^Sc^.^1^ However, the exact mechanism of conversion and the cause of pathogenesis remains unclear.

It is proposed that PrP^Sc^ propagates by a template conversion model, whereby PrP^C^ is converted into further copies of PrP^Sc^ independently of nucleic acids.^2^ Through this mechanism PrP^Sc^ spreads throughout the central nervous system (CNS) of the host, eventually leading to the development of clinical symptoms.^3^ The symptoms themselves are characterized by neuropathy and loss of cognitive function leading to death.^4^ These diseases are classically associated with long asymptomatic incubation periods.^5^

The pursuit of therapeutics for prion diseases has been a research priority for over 2 decades, especially following the BSE epidemic in the United Kingdom from the mid-1980s to 1990s.^6^ This epidemic resulted in the transmission of BSE to humans, leading to the emergence of variant CJD (vCJD).^7^ A recent study has suggested that 1 in 2000 individuals may be carriers of the BSE PrP^Sc^ protein in the UK, highlighting the potential for iatrogenic transmission.^8^ Approaches to the development of therapeutics have included pharmaceuticals, immunotherapeutics, protein and peptide inhibitors, immunopotentiators and many others.^4,6,9^ A number of compounds have even been taken as far as clinical studies, including pentosan polysulphate and quinacrine.^10-12^ However, neither of these compounds have been found to be effective in reducing disease progression in clinically-affected patients.^12-14^

Recently it was reported that heterologous recombinant prion proteins (rPrPs) can act as inhibitors of prion amplification *in vitro* and *in vivo*, highlighting the potential use of rPrPs as therapeutic agents for controlling or slowing the development of prion replication.^15,16^

Here, we use the protein misfolding cyclic amplification (PMCA) assay as an *in vitro* model for prion replication. PMCA is a technique pioneered by Soto and colleagues^17^ and allows the amplification of minute quantities of PrP^Sc^ when seeded into a PrP^C^ substrate and being subjected to rounds of incubation and sonication, this can include the periodic replenishment of PrP^C^ substrate in a process termed serial PMCA (sPMCA).^18^ Using PMCA, rPrPs representing different ovine *Prnp* genotypes were tested as inhibitors of scrapie and BSE replication. In terms of ovine *Prnp* genotype, it is well documented that the VRQ genotype (codons 136, 154 and 171 of *Prnp*, respectively) is associated with high susceptibility to classical scrapie, and ARR is associated with high resistance.^19^ The ARQ genotype is susceptible to classical scrapie and different genotypes may be infected with different prion strains.^20^ The strains that were amplified included ovine TSEs with distinct prion strains, in hosts with distinct *Prnp* genotypes, and replication in PrP^C^ substrate with distinct *Prnp* genotype (combinations are summarised in Table 1).

**TABLE 1.** Prion disease isolates used in this study.

Strain Type	Sample Number	*Prnp* genotype of host	*Prnp* genotype of PMCA substrate
Classical Scrapie	PG1361/05	VRQ/ARQ	VRQ/VRQ
Classical Scrapie	PG1563/02	VRQ/VRQ	VRQ/VRQ
G_338_ Scrapie	MC136477	VRQ/VRQ	VRQ/VRQ
G_338_ Scrapie	MC136553	VRQ/VRQ	VRQ/VRQ
Apl_338_/Apl_338ii_ Scrapie	MC141403	VRQ/VRQ	VRQ/VRQ
Apl_338_/Apl_338ii_ Scrapie	MC141404	VRQ/VRQ	VRQ/VRQ
CH1641 Scrapie	J2935	AHQ/ARQ	AHQ/AHQ
Ovine BSE	PG0392/04	ARQ/ARQ	VRQ/VRQ
Ovine BSE	PG1693/03	ARQ/ARQ	VRQ/VRQ
Bovine BSE	SE1929/0749	Bovine	Bovine
Bovine BSE	SE1945/0035	Bovine	Bovine
Bovine BSE	SE1762/0013	Bovine	Bovine

Classical scrapie and BSE infected brain material were field cases from the APHA (Addlestone, Surrey, UK). BSE samples SE1929/0749, SE1945/0035 and SE1762/0013 were pools of multiple isolates. Ovine BSE samples originated from ovine BSE challenges of sheep (APHA). CH1641 scrapie isolates were a gift from Professor N. Hunter, The Roslin Institute, (University of Edinburgh). Apl_338ii_ and G_338_ scrapie brain tissue was isolated from a transgenic mouse bioassay (transgenic for ovine VRQ *Prnp*).^29^

## RESULTS

**FIGURE 1.**
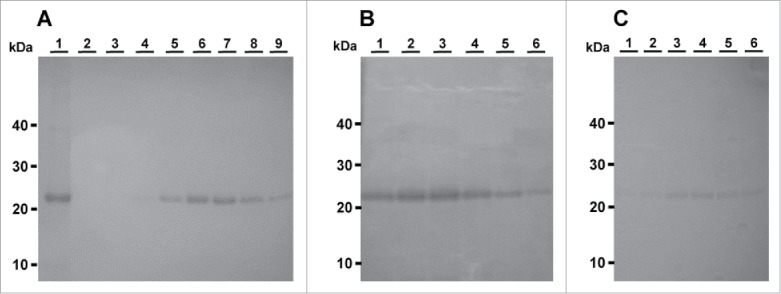
Ovine PrPs rVRQ, rARQ and rARR purified by IMAC. A) Purification of rVRQ. Lane 1: rVRQ from urea solubilised washed inclusion body. Lane 2 to 9: elution fractions. B) Purification of rARQ. Lanes 1-6: elution fractions. C) Purification of rARR. Lanes 1-6: elution fractions. The inclusion body sample was 4.5 μL of a total of 30 mL produced from 1 L of bacterial culture. Each elution fraction is 4.5 μL of 5 mL total volume for each fraction. Molecular mass markers are indicated.

Bacterially expressed recombinant prion proteins were cloned, expressed and purified by immobilised metal affinity chromatography (IMAC) to the level of a single protein band on an SDS-PAGE gel (Fig. 1). Typical yields of the refolded recombinant proteins were 12 mg/L of culture.

**FIGURE 2.**
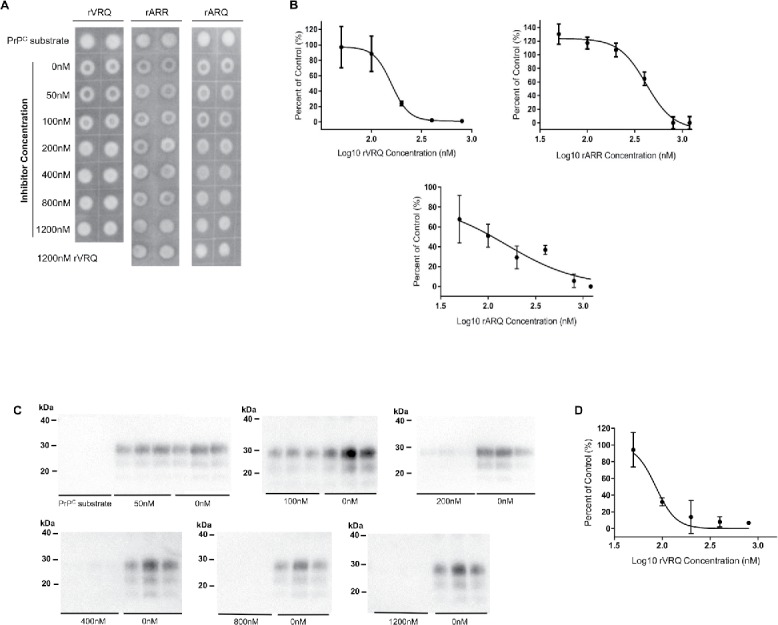
Inhibition of ovine prion replication with distinct rPrPs. A single classical scrapie sample (PG1361/05) was amplified in triplicates by PMCA for one round using a VRQ PrP^C^ substrate. PMCA reaction products amplified in the presence or absence of rVRQ, rARR or rARQ recombinant proteins in a dilution series of 0-1200 nM were each analyzed in duplicate by dot blot (representative blots are shown in A). Protease-resistant PrP^Sc^ was detected with SHa31. Blots were analyzed using ImageJ software and signals expressed as the percentage of the 0 nM inhibitor control signal. Inhibition with 1200 nM of rVRQ was used as a 100% inhibition control and used to calculate the background blot signals. PrP^C^ substrate controls are also shown further illustrating the removal of all PrP^C^ by the PK-digestion procedure. Values were plotted using GraphPad Prism. Inhibition occurred with mean IC50 values of 122 nM for rVRQ, 228 nM for rARQ and 505 nM for rARR calculated from 3 separate experiments (B). All amplification products were also analyzed by western blot (using identical conditions to the dot blots with the exception that 6.7 µL of PK digested PMCA reaction was analyzed) and an example for VRQ inhibition is shown (C), the concentration of rVRQ inhibitor is indicated. All blots also contained analysis, in triplicate, of PrP^C^ substrate that again demonstrated the complete removal of PrP^C^ signals (shown for the first blot only). Densitometry data from the blots is shown (D) and was used to calculate an IC50 value for rVRQ of 85 nM. Molecular mass markers are indicated.

rPrP proteins were assessed as inhibitors within *in vitro* PMCA reactions. TSE samples used are detailed in (Table 1). IC50 values were calculated for rVRQ, rARQ and rARR proteins when inhibiting the replication of a single ARQ/VRQ classical scrapie isolate (Fig. 2). rVRQ was the strongest inhibitor with a mean IC50, when calculated using the dot blot analysis method, of 122 nM, followed by rARQ (IC50 of 288 nM) and rARR (IC50 of 505 nM). The analysis of PMCA products inhibited by 1200 nM rVRQ or where no spike was present demonstrated that the dot blot method was detecting PrP^Sc^ and no residual PrP^C^ signals were present (Fig. 2). Reanalysis of all samples by western blot further demonstrated the specific analysis of PrP^Sc^ and produced IC50 values that gave the same relative efficacy for the rVRQ, rARQ and rARR inhibition: 85, 200 and 515 nM, respectively (Fig. 2 and data not shown).

**FIGURE 3.**
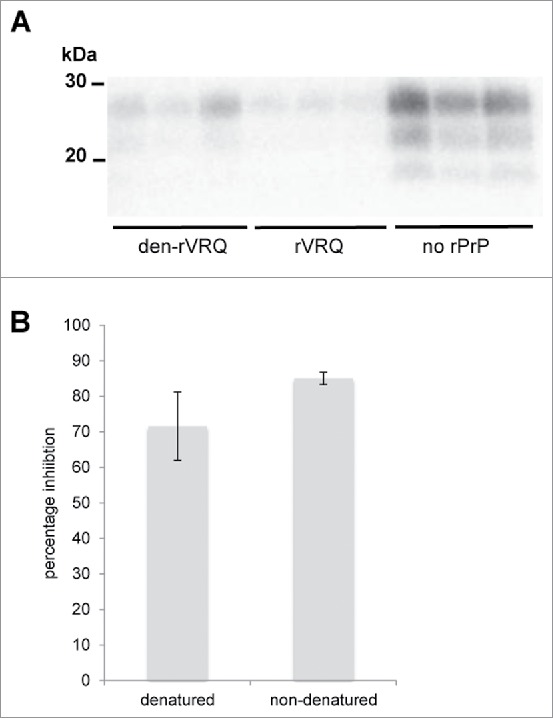
Both native and denatured rVRQ inhibit prion replication. PMCA amplification of a scrapie isolate (PG1361/05) was performed in triplicate in the presence of 1200 nM of rVRQ with or without denaturation or in the absence of any rPrP (as indicated). 6.7 µL of PK digested PMCA reaction was analyzed by western blotting using monoclonal antibody SHa31 (A). Following densitometry, signals were expressed as the percent inhibition, calculated using the no rPrP controls as 100% amplification (B). No significant difference in the inhibition levels between rVRQ and denatured rVRQ was observed (unpaired Student's t-test, p = 0.08). Molecular mass markers are indicated.

It was then determined whether the conformation of the rVRQ was required for inhibition to occur. Heat-denatured rPrP was used at 1200 nM along with non-denatured rVRQ and both inhibited replication of scrapie PrP^Sc^. There was a trend for the denatured rVRQ to show less inhibition but this was not significantly different (p = 0.08; Fig. 3). Addition of a control protein (bovine serum albumin; BSA) to the reactions at 1200 nM did not inhibit replication which was only inhibited by 0.08% (average of triplicate analysis) in the presence of this protein (unpaired Student's t-test analysis gave a p value of 0.98; data not shown).

**FIGURE 4.**
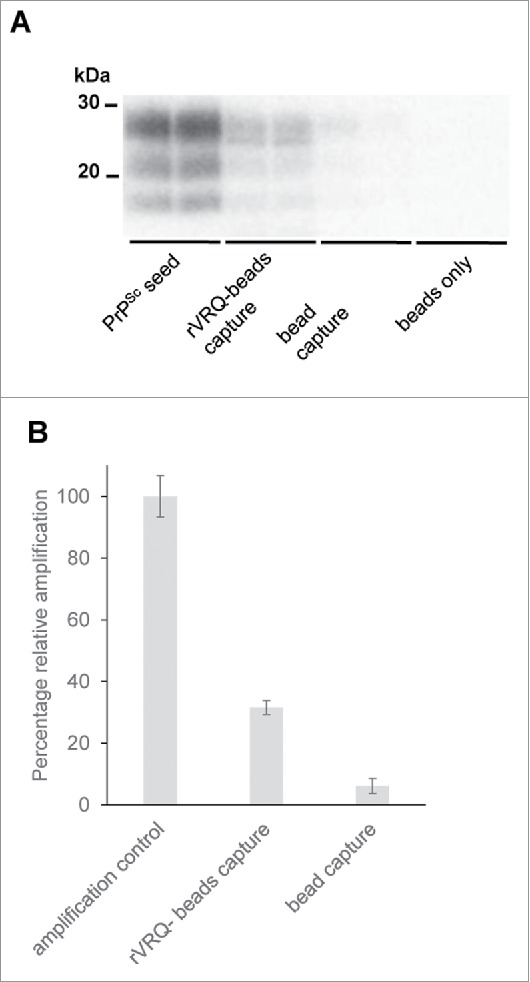
rVRQ binds to PrP^Sc^. Beads were coated with rVRQ (rVRQ-beads capture) or had no rPrP present (bead capture) and were then incubated with brain homogenate from a scrapie-affected sheep (PG1361/05). Following extensive washing, the beads were used to seed PMCA reactions along with beads that had not been incubated with any brain sample (beads only). PMCA products were digested with PK and analyzed for PrP^Sc^ with antibody SHa31 (A). Following densitometry, signals were expressed as the percent amplification compared with a control reaction directly spiked with an equivalent level of PrP^Sc^ (PrP^Sc^ seed) as 100% amplification after the background signals for beads only reactions were removed (B). The difference in PMCA seeding between beads with and without rVRQ was significantly different (unpaired Student's t-test, p = 0.03). Molecular mass markers are indicated.

It was then determined whether rVRQ could bind scrapie PrP^Sc^. After incubation with scrapie-affected brain, beads coated in rVRQ could seed PMCA reactions and this was not the case for beads lacking the recombinant protein (Fig. 4). On average, beads coated in rVRQ captured 32% of the PMCA seeding capability in the brain homogenate, without rVRQ only 6% of seeding activity was recovered.

**FIGURE 5.**
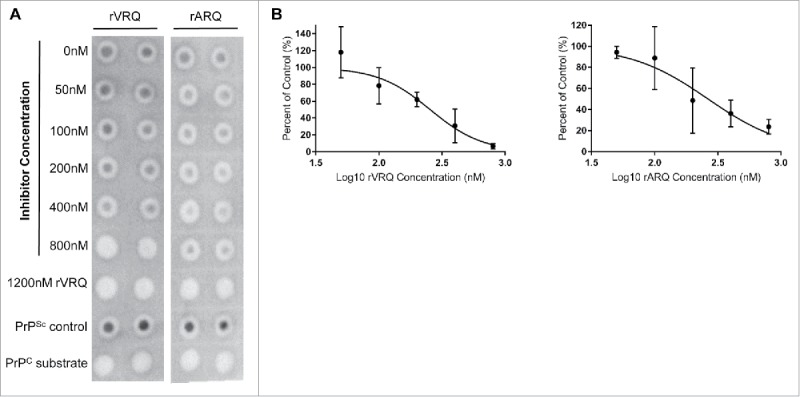
Inhibition of bovine BSE prion replication with rPrPs. Bovine BSE (SE1762/0013) was amplified in triplicates for one round in bovine PrP^C^ substrate. Dot blots of PMCA reaction products amplified in the presence or absence of rVRQ and rARQ in a dilution series of 0-800 nM are shown for representative blots (A). Products were digested with PK and 0.83 µL of the PMCA reaction analyzed by dot blot. PrP^Sc^ was detected with monoclonal antibody SHa31. Blots were analyzed using ImageJ software and signals expressed as the percent of the 0 nM inhibitor control. Inhibition with 1200 nM of rVRQ was used as a 100% inhibition control and used to calculate the background blot signals. A PrP^Sc^ positive control sample and a PrP^C^ substrate were also analyzed on each blot (A). Values were plotted using GraphPad Prism. Inhibition of BSE amplification occurred with IC50 values of 171 nM for rVRQ and 366 nM for rARR calculated from 3 separate experiments (B).

A possible explanation for the greater efficacy of rVRQ as an inhibitor of the replication of this ARQ/VRQ classical scrapie isolate is the homologous sequence between the inhibiting protein, the PrP^Sc^ seed and/or PrP^C^ substrate used for amplification (VRQ/VRQ). To determine whether absolute sequence identity was a requirement for the inhibition of prion replication, the 2 most effective inhibitors as determined by IC50, rVRQ and rARQ, were applied to bovine BSE PMCA amplification (Fig. 5). The mean IC50 values were 171 nM for rVRQ and 366 nM for rARQ.

VRQ rPrP was then tested as an inhibitor of the amplification of isolates over 5 rounds of sPMCA, different scrapie strains were analyzed as well as ovine and bovine BSE. (Table 1).

**FIGURE 6.**
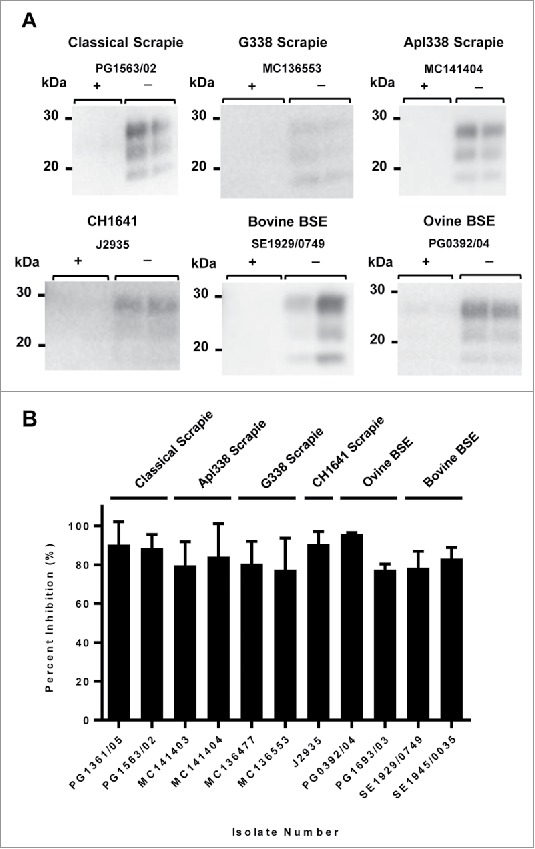
Inhibition of distinct prion strains/isolates with rVRQ. All samples were amplified in duplicate by sPMCA for 5 rounds. Classical scrapie (genotypes VRQ/ARQ and VRQ/VRQ, isolates PG1361/05 and PG1563/02, respectively), transgenic-mouse passaged classical scrapie (2 isolates each of G_338_ and Apl_338_/Apl_338ii_ passaged in mice with genotype VRQ) and experimental ovine BSE (2 isolates, both ARQ/ARQ) were amplified using a VRQ/VRQ PrP^C^ substrate. Experimental CH1641 scrapie was amplified using an AHQ/AHQ PrP^C^ substrate. Two bovine BSE samples were amplified using a bovine PrP^C^ substrate. Representative western blots of PMCA reaction products amplified in the presence (+) or absence (−) of 400 nM rVRQ are shown for each TSE type (A). 6.7 µL of PK digested PMCA reaction was analyzed by western blotting using monoclonal antibody SHa31. Following densitometry, signals were expressed as the percent inhibition, calculated using the no inhibition controls as 100% amplification (B). The data in (B) is collated from 2 separate experiments. Molecular mass markers are indicated.

Scrapie samples included classical ovine scrapie isolates, Apl_338_/Apl_338ii_ and G_338_ scrapie strains derived from tg338 transgenic mice^29^ (both amplified in VRQ substrate), CH1641 scrapie (in an ARQ/AHQ host and amplified in an AHQ substrate), ovine BSE (in an ARQ/ARQ host and amplified in a VRQ substrate) and bovine BSE (amplified in bovine substrate) (Table 1). These samples were chosen because of their efficient amplification over the course of 5 d of sPMCA. PMCA was performed in the presence of rVRQ at 400 nM which consistently inhibited prion amplification (Fig. 6).

## DISCUSSION

Using PMCA as an *in vitro* model for prion replication, ovine PrP was shown to inhibit the replication of VRQ/ARQ scrapie prion in VRQ/VRQ substrate with rVRQ, rARQ and rARR producing mean IC50 values of 122, 288 and 505, respectively (calculated from dot blot analysis of PMCA products). For the inhibition of bovine BSE replication in bovine substrate, the mean IC50 values were 171 nM for rVRQ and 366 nM for rARQ. These data indicate that VRQ was the stronger inhibitor and could inhibit prion replication in the absence of absolute sequence identity with substrate or seed PrP.

Effective concentration 50% (EC50) values have been reported by Yuan *et al.* when analyzing inhibition with rPrP homologous to the seed and substrate PrP sequence.^15^ The models used were for human rPrP23–231 (129V) inhibition of iCJDVV2 (iatrogenic CJD, *PRNP* valine 129 homozygous) amplification in TgWV murine (transgenic for human 129V *PRNP*) substrate, where the approximate EC50 value was 60 nM, and mouse rPrP23–231 inhibition of mouse PrP^Sc^ (strain 139A) amplification in mouse brain homogenate where the approximate EC50 value was 120 nM. These figures are similar to the IC50 values determined in the present study for rVRQ inhibition of homologous genotype seed/substrate.

Here, inhibition was also demonstrated with PrP^Sc^ seeds from bovine and ovine hosts, including ovine hosts with distinct *Prnp* genotypes, and with distinct prion strains, and also when amplification was within substrate with different *Prnp* genotypes (Table 1, Fig. 6). These data indicate that rVRQ may act as a ‘universal inhibitor’ of ruminant prion replication *in vitro*.

The mechanism of rPrP inhibition remains unresolved. In terms of the binding ability of rPrP, Yuan *et al.*^15^ demonstrated that human rPrP23–231 bound to PrP^Sc^ (iCJD) but not PrP^C^, and Meier *et al.* report that murine rPrP binds murine adapted scrapie PrP^Sc^.^21^ The present study also demonstrates that rVRQ bound to ovine scrapie PrP^Sc^ which was then able to seed PMCA reactions. It has been previously suggested that rPrP inhibition is species specific and correlates to sequence similarity.^22^ Here the majority of scrapie strains and ovine BSE used were amplified in a VRQ/VRQ substrate (Table 1) and inhibited with rVRQ. Sequence similarity between inhibitor and seed/substrate may well be important and the most effective inhibition was seen with rVRQ inhibition of seed/substrate with homologous *Prnp* genotype. However, the presented data also indicates that the mechanism for inhibition is not wholly dependent on such sequence similarity. Both rVRQ and rARQ proteins inhibited bovine BSE amplification in bovine brain homogenate substrate, suggesting that the inhibitory activity of these proteins may be independent of sequence similarity. This is further supported by experiments with rVRQ inhibiting different TSE isolates/strains and in prion amplification reactions with substrates of heterologous genotypes. Here, it was also shown that heat denatured rVRQ could inhibit ovine scrapie replication indicating that rPrP conformation is not a major influence on the mechanism of inhibition. It may suggest that internal unstructured PrP amino acid linear sequences are involved in interactions with PrP^Sc^ that facilitate inhibition of prion replication. However, this assumes that incubation of the denatured rPrP at 37˚C during PMCA does not promote full refolding of rVRQ. Using mouse and hamster prion replication models, internal rPrP peptides have previously been shown to inhibit prion replication and such peptides include the region that in sheep contains the 136 polymorphism (hamster PrP residues 109-141).^23,24^ These studies also show that inhibition by this peptide is effective across species where there is not absolute amino acid sequence identity.^24^ This supports other studies using cell-free conversion reactions where heterologous PrP^C^ has been demonstrated to effectively inhibit homologous PrP^C^ to PrP^Sc^ conversion.^25^ If the analogous linear stretch of residues within rPrP described by Chabray and colleagues^23,24^ influences binding of ovine rPrP to ruminant PrP^Sc^ and inhibition of replication, then the 136 polymorphism may influence the efficacy of this process but inhibition of differing PrP^Sc^ primary sequences should be possible, as appears to be the case in the present study.

The inhibitory mechanism of rPrPs may be in part due to the lack of secondary modifications. Different glycosylation states of PrP can selectively affect prion strain amplification.^26^ rPrPs then may act as competitive, but less conversion efficient substrates due to the lack of secondary modifications.^15,16,21^ This may be aided by the concentration of inhibitor used here (400 nM) and by Yuan *et al.*^15^ (200 nM), which are far greater than physiological concentrations of PrP^C^ (∼13 nM in 10% ovine brain homogenate, calculated from the values reported elsewhere^27^). The reported data from an *in vitro* model indicates that rVRQ is a therapeutic candidate that requires examination *in vivo* using transgenic mice. In a recent study, RML-chandler scrapie disease progression in mice was increasingly delayed in response to increasing doses of hamster rPrP. This study suggested that the stoichiometry of host PrP^C^ to rPrP could be an important factor in the inhibitory mechanism and importantly, demonstrated that rPrPs have therapeutic effects *in vivo*.^16^

Why the rVRQ protein acts as an inhibitor of replication for a range of TSE isolates and strains is unexplained, but PrP proteins containing the ovine Q_171_ polymorphism are known to act as superior amplification substrates even to other prion types like BSE and vCJD.^28^ This may indicate that VRQ PrP can effectively interact with PrP^Sc^ from different sources and this may then explain the inhibitory activity across prion types with rVRQ acting as a competitive inhibitor. The reported inhibitory activity of rVRQ highlights the potential application of recombinant PrP proteins as broad-spectrum inhibitors of prion replication. Whether this rVRQ inhibition of ruminant prion replication extends to further host species and prion strains remains to be established.

## MATERIAL AND METHODS

### Samples

Healthy and diseased ovine tissues were obtained from the Animal and Plant Health Agency TSE-Archive (APHA, Addlestone, Surrey, UK). Classical scrapie infected brain material was obtained from scrapie positive specimens submitted to the APHA for testing. Ovine BSE samples originated from ovine BSE challenges of sheep performed by the APHA. The CH1641 scrapie isolate was a gift from Professor N. Hunter, The Roslin Institute, (Neuropathogenesis Division, University of Edinburgh). BSE positive bovine brain samples were pools of 71 BSE positive cases (SE1929/0749 and SE1945/0035) or were a pool of 4 isolates (SE1762/0013), all 3 samples were sourced from the APHA. Apl_338ii_ and G_338_ scrapie brain tissue was derived from a transgenic mouse bioassay (transgenic for ovine VRQ *PRNP*) as described.^29^ All TSE samples used are detailed in Table 1. 10% (w/v) brain homogenates were prepared as described previously.^30^ Healthy ovine brain tissue was obtained from a scrapie-free, New Zealand derived flock (ARSU, APHA) and bovine brain tissue from a confirmed BSE negative Fresian cow.

### Production of Recombinant PrP Proteins

*Prnp* genes for ovine PrP VRQ (23–231) and ARR (23–231) were amplified by PCR from genomic DNA isolated from sheep of known genotype using the primers: Ov PrP pET F 5′-AGAATTCATATGAGCAAGAAGCGTCCAAAACCTGGCGGAGGATG-3′ and Ov PrP pET R 5′-ACTCAGGATCCTATCAACTTGCCCCACGTTGGTAATAAGCCTGGGATTC-3′. Products were digested with NdeI and BamHI and ligated into plasmid pET22b at NdeI and BamHI sites. The sequences of the *Prnp* inserts were confirmed by Sanger sequencing. The construct for the ovine recombinant protein ARQ (23–231) in the expression plasmid pET41 (a+) was obtained from IDEXX Laboratories.

VRQ and ARR recombinant proteins were expressed in Novablue (DE3) *E. coli*, and ARQ recombinant protein was expressed in Top10 *E. coli*. Briefly, bacterial cultures were grown in 2YT containing 100 µg/mL ampicillin (VRQ, ARR) or 50 µg/ml kanamycin (ARQ), and at mid log growth were induced by addition of 1 mM Isopropyl β-D-1-thiogalactopyranoside. Cultures were incubated shaking at 37˚C overnight. Cells were then harvested by centrifugation at 2800 g for 30 minutes and stored at −20˚C before lysis. Cell pellets were resuspended in lysis buffer (50 mM NaH_2_PO_4_, 300 mM NaCl, pH 8.0, 0.1 % Nonidet P-40, 10 mg/mL lysozyme, and Roche complete protease inhibitor without EDTA) and then incubated at 37˚C for 1 hour. DNase I (1 mg/mL, 240 µL) and 120 µL MgCl_2_ (1 M) were added and incubated at room temperature for 15 minutes. Lysate was centrifuged at 12000 g for 20 minutes at 4˚C. The pellets were then resuspended in 25 mL wash buffer (10 mM Tris pH 8.0, 0.1 % Nonidet P-40) and incubated on ice for 20 minutes and pellets recovered by centrifugation as before. Washing was repeated 3 times. Pellets were resuspended in 2.5 mL solubilisation buffer (8 M urea, 50 mM NaH_2_PO_4_, 300 mM NaCl, pH 7.5) overnight at room temperature with rotation. Samples were centrifuged as before and supernatant purified by FPLC using an IMAC Hi-Trap chelating column (5 ml) (GE healthcare) pre-charged with copper sulfate. Protein was refolded on the column as described previously^31^ and then eluted on a 0–0.5 M imidazole gradient in 300 mM NaCl/50 mM NaH_2_PO_4_ (elution buffer). Protein fractions from the elution peak were assessed for purity by SDS-PAGE, staining with Instant Blue (Expedeon). Protein concentrations were determined by Bradford assay against a BSA standard. Protein fractions were pooled and stored at −80°C with 20 % (w/v) sucrose. rPrP was used as an inhibitor directly in PMCA reactions to determine inhibitor concentration 50% (IC50) values for inhibition of the classical scrapie PG1361/05. For subsequent PMCA experiments, imidazole and sucrose were removed from protein preparations by 2 rounds of dialysis against PMCA conversion buffer (50 mM NaCl, 4 mM EDTA (pH 8), 1% (v/v) Triton X-100, pH 7.4). Protein concentrations were determined by Bradford assay.

### Protein Misfolding Cyclic Amplification

PMCA reactions were performed as described previously.^32^ Briefly, substrate, PrP^Sc^ “spike” and rPrP were combined in clear 0.2 mL PCR tubes (Corning). PMCA was performed at 37˚C at a power setting of 190-200 W in an ultrasonicating water bath (model 3000; Misonix). Sonication was performed for 40 seconds followed by 29 minutes and 20 seconds of incubation, this cycle was repeated for 24 hours. For serial PMCA (sPMCA) experiments, 50 µL of reaction products were then added to 100 µL of fresh substrate (including appropriate inhibitors) and 100 µL of this was used in the subsequent round of PMCA. Reaction products were stored at −20˚C before analysis. Reactions to determine relative IC50 values were prepared with 88 µL of brain homogenate substrate and rPrP to a total volume of 95 µL. For amplification of PG1361/05 only, sucrose was added to a final concentration of 2% (w/v). The concentration of each rPrP was adjusted before use by dilution in elution buffer. PrP^Sc^ “spike” (5 µL) was then added. Reactions were performed in triplicates and run for a single round of PMCA. IC50 values were determined for amplification of a classical scrapie isolate (PG1361/05) and a bovine BSE isolate (SE1762/0013). For scrapie amplification, concentrations of rPrPs of 50 nM, 100 nM, 200 nM, 400 nM, 800 nM and 1200 nM were analyzed with amplifications of 1% (w/v) scrapie brain homogenate in VRQ/VRQ brain homogenate substrate. Controls included uninhibited PMCA reaction controls without any inhibitor present and reactions treated with 1200 nM rVRQ as 100% inhibition controls. For bovine BSE amplification, PMCA was performed using 10% (w/v) BSE brain homogenate (due to lower amplification efficiencies) in bovine brain homogenate substrate and a dilution series of 50 nM, 100 nM, 200 nM, 400 nM and 800 nM recombinant proteins. All triplicates were each analyzed by dot blot in duplicate and IC50 values were then calculated using the mean value for each inhibitor concentration. The experiments to determine IC50 values were repeated 3 times and the mean IC50 value for each inhibitor across all experiments reported. Western blot analysis of some of the samples was also performed, analyzing triplicate sPMCA reactions for each inhibitor concentration and the IC50 value determined using a mean values for each condition.

When assessing the inhibition of scrapie isolate PG1361/05, PMCA reactions were also performed in triplicate in the presence of 1200 nM of BSA, non-denatured rVRQ or rVRQ denatured by heating to 100°C for 10 minutes and cooling immediately on ice.

Dialysed VRQ was also tested as an inhibitor of the amplification of a range of isolates. Reactions were prepared as described above using 400 nM of VRQ rPrP only. Reactions were spiked with 5 µL of 1 % (w/v) brain homogenate for each isolate, except for CH1641 isolate J2935, and bovine BSE samples SE1929/0749 and SE1945/0035 which were spiked at 5 µL 10% (w/v) homogenate due to lower amplification efficiencies. All reactions were performed in duplicate for 5 rounds of sPMCA. For PrP^Sc^ detection, 20 µL of PMCA product was digested with PK and 6.7 µL of PMCA reaction analyzed by western blotting.

### Capture of PrP^Sc^ with rVRQ

rVRQ was immobilised to tosylactivated magnetic beads following the manufacturer's instructions (Invitrogen). Briefly, 10 mg of beads were reacted with 200 μg of rVRQ in 0.1 M borate buffer overnight at 37°C with shaking. Beads were washed 3 times in TBS and incubated with 33.5 μl of 10% (w/v) brain homogenate PG1361/05 for 1 hour at room temperature with rotation. Beads were then washed 5 times in TBS. An equivalent experiment was performed without any rVRQ. Beads (150 μg) from these 2 experiments along with beads that had not been incubated with brain homogenate were then used to spike duplicate PMCA reactions. PrP^Sc^ amplification was compared with reactions spiked directly with 5 μl of 1% (w/v) brain homogenate from PG1361/05.

### Western Blots and Dot Blots

PMCA reaction products were digested with 50 µg/mL proteinase K (PK), 0.045% (w/v) SDS for 1 hour at 37°C and then boiled in LDS loading buffer (Invitrogen). PMCA product (0.83 µL) was spotted onto nitrocellulose, blocked with 3% (w/v) milk powder and probed with the monoclonal antibody SHa31 at a dilution of 1:40000 in 0.5% (w/v) milk powder and 0.5% (v/v) tween 20 in TBS buffer (50 mM Tris-HCl pH 8, 150 mM NaCl) (TBST). Bound antibody was detected with polyclonal goat anti-mouse-horseradish peroxidase (HRP) (Dako) at a dilution of 1:20000 in 0.5% (w/v) milk powder TBST. Membranes were then incubated with HRP substrate from an EZ-Chemiluminescence Detection Kit (Geneflow) and signals detected using a Photek Photon Counting System. Images were analyzed using ImageJ software.^33^ 1200 nM rVRQ treated reactions were measured as background readings and subtracted from the other signals. Percent inhibition values were then calculated relative to the mean intensity of uninhibited control reactions. Calculated values were plotted using GraphPad Prism and IC50 values were determined by nonlinear regression, log inhibitor verses response (variable slope) model with constraints at 100% and 0%. This model calculated the IC50 value from the data and did not assume a sigmoidal relationship. Some data sets were reanalysed by western blots as described previously^30^ and analyzing 6.7 µL of PMCA product. Prion detection steps and densitometry analysis were as for dot blots.

## ABBREVIATIONS

BSE: Bovine Spongiform Encephalopathy
CJD: Creutzfeldt Jakob Disease
PMCA: Protein Misfolding Cyclic Amplification
PrP: Prion Protein
rPrP: recombinant PrP
TSE: Transmissible Spongiform Encephalopathy
